# Treatment of Primary Aldosteronism and Organ Protection

**DOI:** 10.1155/2015/597247

**Published:** 2015-05-17

**Authors:** Cristiana Catena, GianLuca Colussi, Leonardo A. Sechi

**Affiliations:** Hypertension Unit, Internal Medicine, Department of Experimental and Clinical Medical Sciences, University of Udine, 33100 Udine, Italy

## Abstract

Primary aldosteronism is a frequent form of secondary hypertension that had long been considered relatively benign. Experimental and clinical evidence collected in the last two decades, however, has clearly demonstrated that this endocrine disorder is associated with excess cardiovascular and renal complications as compared to essential hypertension. These complications reflect the ability of inappropriate elevation of plasma aldosterone to cause tissue damage beyond that induced by high blood pressure itself, thereby setting the stage for major cardiovascular and renal disease. Because of the impact of elevated aldosterone on organ damage, goals of treatment in patients with primary aldosteronism should not be limited to normalization of blood pressure, and prevention or correction of organ complications is mandatory. Treatment with mineralocorticoid receptor antagonists or unilateral adrenalectomy is the respective options for treatment of idiopathic adrenal hyperplasia or aldosterone-producing adenoma. Last years have witnessed a rapid growth in knowledge concerning the effects of these treatments on cardiovascular and renal protection. This paper is an overview of the cardiovascular and renal complications that occur in patients with primary aldosteronism and a summary of the results that have been obtained in the long term on cardiovascular and renal outcomes with either medical or surgical treatment.

## 1. Introduction

Aldosterone is secreted by the outermost portion of the adrenal cortex and contributes to regulation of blood pressure. Aldosterone acts at the distal tubular site of the nephron where it increases water and sodium reabsorption thereby leading to extracellular fluid expansion. Evidence collected in the last two decades indicates that, in addition to the regulatory role in body fluid and electrolyte balance, aldosterone affects a variety of cell functions that may result in tissue fibrosis. Expression of mineralocorticoid receptors has been detected in different cell types obtained from cardiac and vascular tissues [1] and robust experimental evidence indicates that prolonged exposure to inappropriately elevated concentrations of aldosterone causes cardiac and renal damage independent of blood pressure levels [2].

Primary aldosteronism (PA) is one of the most frequent forms of secondary hypertension and among patients with high blood pressure there is evidence of a much higher prevalence of this condition than the previously estimated frequency of less than one percent [3–5]. Hypertension due to primary aldosteronism had long been considered a form of disease associated with a relatively low incidence of cardiovascular and renal complications [6, 7]. This could be explained by the suppression of renin activity and the related decrease of angiotensin II formation [8]. More recent views, however, indicate that a variety of cardiovascular and renal sequelae that are not merely due to elevated blood pressure are associated with PA and make this condition far from being benign [9].

Idiopathic adrenal hyperplasia (IHA) and aldosterone secreting adrenal adenoma (APA) account for more than 95% of forms of PA and, with few exceptions, are characterized, respectively, by bilateral or unilateral involvement of the adrenal glands [10]. Administration of mineralocorticoid receptor (MR) antagonists or unilateral adrenalectomy is the respective options for treatment, as indicated in consensus documents [11]. Both treatments are effective in reducing blood pressure, although in a relevant proportion of patients using antihypertensive drugs is needed after treatment to obtain blood pressure normalization [12]. Moreover, because of the impact of elevated aldosterone on organ damage, goals of treatment in patients with PA should not be limited to normalization of blood pressure and hypokalemia and prevention or correction of organ complications becomes mandatory. In fact, substantial evidence obtained in recent years indicates that cardiovascular and renal outcomes of patients with PA do benefit from both MR antagonists and surgical treatment, although the relative efficacy of these treatments in the long term remains under debate [13]. This issue will need further investigation because reversal of aldosterone-related tissue damage is the main factor that could justify the efforts and expenses of procedures that are used to differentiate unilateral or bilateral adrenal disease. Ongoing debate has been generated upon this subject due to different views of some opinion leaders who claim the opportunity for search of lateralized aldosterone secretion by adrenal venous sampling in all PA patients [14, 15] and others who would limit this procedure to selected groups of patients [16, 17].

The last few years have witnessed an explosive growth in knowledge of the genetic basis of PA, and many different somatic mutations, all involving genes encoding ion channels, have been described in patients with APAs [18]. After the first seminal report of Choi et al. [19] in which two different mutations (G151R and L168R) were identified in Kir 3.4 (KCNJ5), an inwardly rectifying potassium channel, other somatic mutations of the alpha-subunit of Na^+^/K^+^ ATPase (ATP1A1) [20], Ca^2+^ ATPase calcium channel (ATP2B3) [21], and voltage-gated calcium channel (CACNA1D) [22] have been demonstrated in APAs removed from PA patients. Their relative prevalence has subsequently been characterized in patients with PA of different ethnicity [23, 24]. Detection of these mutations has been associated with some features of patients with PA including more severe aldosterone overproduction [19, 24] or reduced responsiveness of plasma aldosterone to postural changes [23]. Therefore, it might be possible that patients with APAs carrying mutated genes have worse responses to treatments and possibly a greater burden of cardiovascular and renal complications, both issues that start being investigated in specifically designed studies [25]. This paper is an overview of the cardiovascular and renal complications that occur in patients with PA and a summary of the results that have been obtained in the long term on cardiovascular and renal outcomes with either medical or surgical treatment.

## 2. Cardiovascular Outcomes

As stated previously, current treatment for APA is adrenalectomy, because surgery apparently confers a greater possibility of cure and avoids the possible side effects of MR antagonists [26]. Chronic administration of these agents, however, is the treatment of choice in IHA. Although primary aldosteronism is considered correctable, in many cases, hypertension may persist after treatment [27–29]. Most studies on the effects of treatment of primary aldosteronism on blood pressure have been conducted in patients with APA, and a cumulative analysis of initial case series indicated a rate of hypertension cure of 59% following adrenalectomy [30]. In most of these series, however, cure was defined by reaching a blood pressure of less than 160/100 mm Hg. Subsequent evidence has indicated that only approximately one third of patients treated for PA reach a blood pressure of less than 140/90 mm Hg without the use of additional antihypertensive drugs [5, 30, 31]. These estimates were obtained in retrospective investigations and are in full agreement with the results of a prospective study of PA patients with either IHA or APA, 39% of whom had their blood pressure normalized by spironolactone or adrenalectomy, respectively, while in the remaining 61% blood pressure levels fell by more than 20% and fewer antihypertensive agents were needed to reach values below 140/90 mm Hg [28].

Initial studies reported a low prevalence of cardiac complications in patients with PA [6] and subsequent cross-sectional assessments yielded evidence of a prevalence of such complications comprised of 14% to 35% [4, 32, 33]. Most of these prevalence studies, however, had important limitations due to lack of appropriate control subjects. In a first longitudinal retrospective study [34], a significantly increased rate of cardiovascular events was reported in patients with PA as compared to patients with essential hypertension (EH). The study reported a striking increase in the relative risk of myocardial infarction (6.5), atrial fibrillation (12.1), and stroke (4.2). We conducted a study [35] in 54 patients with PA and 323 patients with EH who were accurately matched for age, gender, severity and estimated duration of hypertension, and cardiovascular risk scores. Comparisons revealed a greater prevalence of cardiovascular disease in patients with PA than in those with EH, with an odds ratio of 2.80, 4.93, and 4.36 for coronary heart disease, sustained arrhythmia (mostly atrial fibrillation), and cerebrovascular events, respectively. In this study, prevalence of cardiovascular complications was comparable in patients with idiopathic or tumoral adrenal disease, showing that both subtypes of PA are at increased risk. In the cohort of the German Conn's registry [36], prevalence of coronary heart disease was 16.3%, and that of atrial fibrillation and ventricular arrhythmias was 7.1% and 5.2%, respectively. Although this survey did not have a control group, prevalence of cardiac events in patients with PA was much higher than that of registry patients with EH and comparable cardiovascular risk. More recently, prevalence of cardiovascular events has been compared in 459 patients with PA and 1290 matched patients with EH [37] confirming significantly higher prevalence rates of myocardial infarction, atrial fibrillation, and heart failure. Finally, in a longitudinal retrospective comparison of 270 patients with PA with 1 : 3 case-control matched patients with EH, Mulatero et al. [38] reported a higher rate of cardiovascular events including stroke and arrhythmia over a median follow-up of 12 years. Consistent with our previous findings, these authors reported comparable rate of events in PA patients with IHA or APA. Thus, findings of all these studies convincingly demonstrate that elevated aldosterone levels in PA contribute to cardiovascular damage independent of blood pressure (Table 1). To further support this conclusion, prospective evidence has linked, in the long term, treatment of PA with improvement of cardiovascular outcomes [35]. These outcomes were compared in patients with EH and patients with PA who had comparable cardiovascular risk profile but greater retrospective incidence of coronary heart disease, arrhythmias, and cerebrovascular events. Patients were followed for an average period of 7.4 years after treatment, during which occurrence of a combined cardiovascular endpoint was comparable in the two groups. Analysis of patients with PA treated with MR antagonists or adrenalectomy did not reveal significant differences in cardiovascular outcome, indicating that medical and surgical treatment are equally effective in the prevention of cardiovascular events. In a more recent prospective study, the cardiovascular risk has been compared in 132 patients with EH and 102 patients with PA who had worse baseline risk profile and were treated with either MR antagonists or adrenalectomy [39]. After an average follow-up of 44 months, the cardiovascular risk of PA patients was significantly reduced and became comparable to that of patients with EH.

It is known that subclinical cardiac and vascular changes anticipate major cardiovascular events in patients with high blood pressure. In patients with PA, higher prevalence of a variety of cardiac abnormalities has been reported together with structural [40] and functional [41] vascular changes. Left ventricular hypertrophy is a strong and independent risk factor for major cardiac events [42] and cross-sectional echocardiographic studies reported greater left ventricular mass and more frequent left ventricular hypertrophy in patients with PA than patients with other types of hypertension. Moreover, detection of inappropriately elevated left ventricular mass is increased in PA even in the absence of left ventricular hypertrophy [43] suggesting that in high aldosterone states left ventricular mass increases beyond the amount needed to compensate for the blood pressure related workload. Also, in PA excess left ventricular mass is associated with abnormal left ventricular filling suggestive of diastolic dysfunction and myocardial fibrosis [44]. A magnetic resonance imaging study demonstrated increased myocardial fibrosis and ventricular stiffening in 25 patients with PA as compared to matched normotensive controls [45] and plasma procollagen levels were found to be higher in 20 patients with APA as compared to patients with EH, a difference that disappeared after adrenalectomy [46].

Echocardiographic investigations of cardiac changes after treatment of PA were initially confined to short-term studies performed after adrenalectomy and showed significant reduction of the left ventricular mass together with improvement of diastolic function [44]. Later on, data on long-term effects on cardiac structure and function of both medical and surgical treatment of PA have been published [47]. In a first 7-year follow-up study, it was demonstrated that PA patients treated with either spironolactone or adrenalectomy have significant and comparable decrease in left ventricular mass, although decrease is significant within the first year only after surgery [28]. More recently, two studies [48, 49] have reported a decrease of left ventricular mass both in medically and surgically treated PA patients, but changes have reached statistical significance only in the latter group, whereas another study did not find any change in left ventricular mass in patients treated with MR antagonists [50]. These studies have been the subject of a recent meta-analysis [51] indicating that the effects on left ventricular mass of medical and surgical treatment of PA do not differ significantly (Table 2). Finally, in a multivariate regression analysis of 54 patients with PA who were followed for 6.4 years after treatment with spironolactone or adrenalectomy it was reported that the degree of reduction in left ventricular mass in PA patients treated both medically and surgically was independently predicted not only by change in blood pressure but also by pretreatment plasma aldosterone levels [52]. This observation further supports the hypothesis that aldosterone contributes to development of left ventricular hypertrophy independent of the hypertension-related hemodynamic load. Because of recent advances in understanding the genetic basis of PA, future efforts will have to address that possible relevance of somatic mutations detected in APAs for cardiovascular complications of PA and outcomes of treatment. In a recent study of 129 patients with APA, Rossi et al. have reported that plasma aldosterone levels and left ventricular mass are higher in patients with APA carrying KCNJ5 mutations as compared to patients with wild-type APA [25]. However, in this study no differences in left ventricular mass reduction were observed after adrenalectomy in patients with mutated or wild-type APA.

High-affinity MR has been detected in cardiac myocytes and fibroblasts [1] and their activation has been shown to induce myocardial damage independent of blood pressure elevation [2]. Animal experiments demonstrated that aldosterone infusion causes myocardial fibrosis in rats that are fed a high-salt containing diet [53]. In these animals, fibrosis is preceded by inflammation of the perivascular tissue [54] and both myocardial inflammation and fibrosis can effectively be prevented by either removal of adrenal glands or administration of MR antagonists [55]. Activation of MR might therefore cause myocardial changes and the related cardiac dysfunction through a variety of mechanisms including activation of oxidative stress [56, 57]. In fact, the beneficial effects on myocardial fibrosis of MR antagonists [58] have been associated with decreased generation of reactive oxygen species in cardiomyocytes [59] and additional evidence of an involvement of mechanisms that are tightly related to induction of oxidative stress has been obtained in genetically manipulated animals [60, 61]. Interruption of MR-mediated mechanisms might explain why, in the long term, treatment of primary aldosteronism with MR antagonists has comparable effects to the removal of APA in reducing left ventricular mass although this response occurs later than removal of excess circulating aldosterone by adrenalectomy. Persistent hyperaldosteronemia with possible involvement of nongenomic effects of aldosterone [62] might possibly explain the difference in response to medical or surgical treatment.

MRs are found in epithelial and nonepithelial tissues with high affinity for aldosterone and glucocorticoid hormones. Under physiological conditions, the majority of MRs in nonepithelial tissues are bound to cortisol, whereas in epithelial tissues, binding of cortisol to MR is prevented by 11*β*-hydroxysteroid dehydrogenase (11*β*-HSD2), the enzyme that converts cortisol to the receptor-inactive cortisone. Moreover, activity of 11*β*-HSD2 generates NADH from NAD and produces changes in the intracellular redox potential that might, in turn, inactivate the glucocorticoid-MR complex [63]. 11*β*-HSD2 is not present in nonepithelial tissues including the heart, but in such tissues, changes of the intracellular redox potential can result from generation of reactive oxygen species and thereby affect the activity of the MR [63].* In vitro* experiments have demonstrated that changes in the redox potential of cardiomyocytes by exposure to oxidized glutathione turn cortisol from being an MR antagonist to an agonist [64]. Recently, it has been shown that aldosterone itself induces changes in the intracellular redox potential in different cell types [65] through an activation of the NOX1 catalytic subunit of NAD(P)H oxidase. This aldosterone-dependent change in the redox potential is amplified by exposure to high concentrations of salt [65] leading to increased production of reactive oxygen species and thereby to cellular and tissue injury.

## 3. Renal Outcomes

Although animal studies demonstrated that inappropriately elevated aldosterone levels for the salt status cause renal tissue damage [66], the clinical evidence of a role of aldosterone as a potential contributor to renal dysfunction is weaker than that emerged for the cardiovascular system [67]. Indirect evidence of untoward renal effects of aldosterone came from demonstration of benefits obtained with use of MR antagonists in patients with proteinuric disease due to diabetes [68] or other types of renal disease [69]. Cross-sectional studies of renal function in PA have reported high variability in prevalence of renal damage [32]. Initial studies with kidney biopsies demonstrated only moderate tissue damage in patients with PA and reported prevalence of impaired renal function in no more than 7% of patients with this condition [70]. Similarly, a single-center study has reported 24-hour creatinine clearance of less than 60 mL/min/1.73 m^2^ in only 7% of 56 patients with PA [71], whereas in the German Conn's Registry increased plasma creatinine concentration was found in a substantially higher percentage of patients [72]. In patients with PA prevalence of overt proteinuria varied from 8% [60] to 24% [70] a disparity that could be explained by differences in severity and duration of hypertension. In a large, multicenter, Italian study [73], prevalence of microalbuminuria in patients with PA was twofold that of patients with EH.

Crucial data on the role of the kidney in PA were reported in two prospective studies in which PA patients were followed in the short and long term after treatment. In 25 patients with adrenal adenoma who were followed for 6 months after adrenalectomy, Ribstein et al. [74] found a significant decrease in urinary protein excretion that was associated with a significant decrease in glomerular filtration. In a 9-year follow-up study of patients with either IHA or APA, we demonstrated that microalbuminuria is more likely to subside to normal levels after treatment than to progress to overt proteinuria [75]. In this study, normalization of urinary protein excretion was more frequent in patients with PA than in matched patients with EH and this effect was independent of blood pressure changes. Renal outcomes were assessed by measuring the rates of change of glomerular filtration rate and urinary protein excretion. After an initial fall in creatinine clearance observed in patients with PA, subsequent declines in glomerular filtration in patients with PA (−1.15 mL/min/1.73 m^2^/year) or EH (−1.06 mL/min/1.73 m^2^/year) were comparable. Urinary albumin losses did not differ between patients with PA or EH during the long-term phase of follow-up. Separate analysis of renal outcomes in patients with PA who were treated with spironolactone or adrenalectomy did not reveal significant differences.

These two important prospective studies have consistently indicated that PA is characterized by a partially reversible renal dysfunction, suggesting that proteinuria might be the marker of a renal hemodynamic defect. Consistent with the findings of past investigations conducted on the effects of mineralocorticoid hormones on renal function [76] some of which in experimental animals with use of the renal micropuncture technique [77], these two studies have demonstrated the presence of relative glomerular hyperfiltration in patients with PA as compared with appropriately matched patients with EH. The analysis of the patients included in the German Conn's Registry [72] has confirmed that glomerular filtration falls rapidly after treatment of PA and remains relatively stable thereafter. Also and most importantly, evaluation of intrarenal Doppler velocimetric indexes has demonstrated significantly lower intrarenal vascular resistance in patients with PA as compared to patients with EH and reversal of this abnormal intrarenal hemodynamic pattern one year after treatment [78]. In summary, findings of longitudinal studies consistently demonstrate that the hallmark of renal dysfunction in PA is reversible glomerular hyperfiltration that is caused by reduced intrarenal vascular resistance and increases urinary protein excretion. The frequency of regression of microalbuminuria in PA suggests that urinary albumin excretion is, at least in part, a marker of functional rather than structural renal changes. On the other hand, long-term persistence of albuminuria in a substantial proportion of patients with PA is associated with detectable baseline plasma renin levels [27, 71] suggesting coexistence of structural intrarenal vascular damage presumably due to long-standing hypertension prior to treatment.

Similar to the heart, exposure to aldosterone levels that are inappropriate for the salt status or activation of MR can damage the kidney via mechanisms that are independent of blood pressure [2]. Elevated aldosterone produces intrarenal vascular injury, glomerular damage, and tubulointerstitial fibrosis in the kidney of stroke-prone spontaneously hypertensive [79] and uninephrectomized rats [80]. Because of the relationship between aldosterone-related tissue damage and salt intake, it was suggested that effects of high-salt intake could be dependent on activation of MR and that this activation might reflect increased oxidative stress [81]. In fact, infusion of aldosterone in the presence of high-salt diet increases the expression of the renal nicotinamide adenine dinucleotide phosphate [NAD(P)H]-oxidase 4 (NOX4), increasing the generation of reactive oxygen species in the kidney. In these high-salt fed animals, administration of MR antagonists or NAD(P)H-oxidase inhibitors prevents aldosterone-induced increase in blood pressure and reduction of plasma nitric oxide levels and decreases urinary markers of oxidative stress [82]. In summary, in addition to the well-known effects of salt excess on vascular stiffening and blood pressure increase, some effects of salt loading might depend on MR activation reflecting increased oxidative stress.

Another issue that is worth mentioning is related to prevalence of simple renal cysts in PA. In a first cross-sectional study, Torres et al. [83] used CT scans to assess presence, number, and total volume of simple cysts in kidneys of patients with PA in comparison to EH. Renal cysts were present in 24 of 55 patients with PA with a prevalence of 44% versus a prevalence of 25% in age-matched patients with EH. Also, in this study a higher overall renal cysts number and volume were found in patients with PA. In other studies without appropriate control groups, prevalence of renal cysts in patients with PA varied from 16% to 26% [27, 84, 85] depending upon differences in age, sex distribution, duration of hypertension, severity of hypokalemia, and methods used to detect cysts. In a prospective study, we have examined the prevalence of renal cysts in a group of patients with PA who were reassessed after a median follow-up of 6.2 years [86]. At baseline, patients with PA (both IHA and APA) were compared with age and sex-matched patients with EH and normotensive controls. The prevalence of renal cysts was significantly greater in patients with PA (37%) than in patients with EH (18%) and normotensive controls (12%) as well as the average number of renal cysts per patient (1.11, 0.51, and 0.25, resp.). Also and most importantly, development of renal cysts subsided after medical or surgical treatment of PA and no significant change of cyst number and cyst total volume from baseline was found at the end of study in both IHA and APA. Thus, renal cystic disease is highly prevalent in PA and treatment halts its progression, supporting the contention that persistent hypokalemia is the causative factor.

## 4. Conclusions

Current evidence unquestionably indicates a high rate of cardiovascular complications in patients with PA, showing that this condition is far from being benign. The high rate of complications in PA patients, however, is not merely explained by blood pressure elevation. Elevated aldosterone causes myocardial hypertrophy and fibrosis over that induced by high blood pressure itself and, together with vascular structural and functional changes, sets the stage for future cardiovascular events. With regard to the kidney, there are PA intrarenal hemodynamic changes leading to relative glomerular hyperfiltration and increased urinary protein losses. These functional changes are fully reversible until structural damage of intrarenal vessels ensues as the result of persistent hypertensive insult. Follow-up studies of patients with PA indicate that both medical and surgical treatments effectively lower blood pressure, correct subclinical organ damage, and decrease the risk of cardiovascular events and renal disease progression. In this view, timely identification of PA patients becomes mandatory to effectively prevent organ complications. Although during the last years many questions related to the effects of aldosterone on the cardiovascular and renal system have been satisfactorily answered, many others still await clarification.

## Figures and Tables

**Table 1:** Relative risk of cardiovascular events in patients with primary aldosteronism versus patients with essential hypertension.

Study	Coronary heart disease	Cerebrovascular disease	Sustained arrhythmia
Milliez et al. 2005 [34]	6.5	4.2	12.1
Catena et al. 2008 [35]	2.8	4.4	4.9
Savard et al. 2013 [37]	2.6	—	5.0
Mulatero et al. 2013 [38]	0.9	2.2	2.2

**Table 2:** Summary of results obtained in studies that evaluated the long-term response of left ventricular mass to either medical treatment or surgical treatment in primary aldosteronism.

Study	Follow-up years	Medical treatment		Surgical treatment	
		Baseline	End of study	Baseline	End of study
Catena et al., 2007 [28]	6.4	52 ± 2	44 ± 2^†^	53 ± 2	43 ± 2^∗^
Giacchetti et al.^∗^ 2007 [49]	3.7	135 ± 4	125 ± 7	126 ± 5	116 ± 5^∗^
Bernini et al., 2012 [50]	2.7	65 ± 2	65 ± 3	58 ± 2	49 ± 3^∗^
Rossi et al., 2013 [48]	3.0	50 ± 1	47 ± 1	53 ± 1	49 ± 1^∗^

Values of left ventricular mass index are expressed as g/m^2.7^ except for the study of Giacchetti et al. (∗) that expressed as left ventricular mass (g). ^†^ *P* < 0.05 versus baseline.

Data in the table have been included in a meta-analysis [51] showing that effects of medical and surgical treatment of primary aldosteronism on left ventricular mass are not significantly different.
